# Improved Housing Accessibility for Older People in Sweden and Germany: Short Term Costs and Long-Term Gains

**DOI:** 10.3390/ijerph14090964

**Published:** 2017-08-26

**Authors:** Björn Slaug, Carlos Chiatti, Frank Oswald, Roman Kaspar, Steven M. Schmidt

**Affiliations:** 1 Department of Health Sciences & Centre for Ageing and Supportive Environments (CASE), Lund University, SE-221 00 Lund, Sweden; carlos.chiatti@med.lu.se (C.C.); steven.schmidt@med.lu.se (S.M.S.); 2 Interdisciplinary Ageing Research, Faculty of Educational Sciences, Goethe University, 60323 Frankfurt am Main, Germany; oswald@em.uni-frankfurt.de; 3 Cologne Center for Ethics, Rights, Economics, and Social Sciences of Health, University of Cologne, 50923 Cologne, Germany; roman.kaspar@uni-koeln.de

**Keywords:** ageing society, ADL, housing adaptations, environmental barriers, simulations

## Abstract

The physical housing environment is important to facilitate activities of daily living (ADL) for older people. A hindering environment may lead to ADL dependence and thus increase the need for home services, which is individually restricting and a growing societal burden. This study presents simulations of policy changes with regard to housing accessibility that estimates the potential impact specifically on instrumental activities of daily living (I-ADL), usage of home services, and related costs. The models integrate empirical data to test the hypothesis that a policy providing funding to remove the five most severe environmental barriers in the homes of older people who are at risk of developing dependence in I-ADL, can maintain independence and reduce the need for home services. In addition to official statistics from state agencies in Sweden and Germany, we utilized published results from the ENABLE-AGE and other scientific studies to generate the simulations. The simulations predicted that new policies that remove potentially hindering housing features would improve I-ADL performance among older people and reduce the need for home services. Our findings suggest that a policy change can contribute to positive effects with regard to I-ADL independence among older people and to a reduction of societal burden.

## 1. Introduction

The acceleration of demographic ageing is one of the main challenges for European policy-making. As the baby-boom generation retires, the EU’s employed population will keep shrinking while the number of people aged over 60 will increase by about two million every year [1]. The combination of a smaller working population and a higher share of retired people will put additional strains on European welfare systems.

Filling the gap for long-term care demand is today one of the main priorities of the European Union. The policy responses identified to tackle this challenge are raising the efficiency and effectiveness of care delivery; reducing the incidence and prevalence of frailty and disability; and enabling older people to continue to manage independent living with functional limitations (e.g., within adaptable private homes).

In this regard, the physical housing environment represents a key factor to facilitate activities of daily living (ADL) for older people [2,3]. A hindering environment may lead to ADL dependence and thus increase the need for home services, which is individually restricting and a growing societal burden [4]. In order to reduce environmental demand and increase people’s ability to live independently, housing adaptations (HA) are currently provided in several countries to users with various levels of competence loss or clinical conditions. HA can be defined as those “alterations of permanent physical features in the home and the immediate outdoor environment” [5]. The term HA is being used interchangeably with the expression ‘home modification’ (HM), although in contexts such as the U.S., HM includes a wider range of interventions such as assistive technologies and ADL training [6]. According to a recent national report on HA/HM in Sweden, the most common measures concerned thresholds, grab bars, ramps, and adaptation of hygiene areas [7]. HA/HM most commonly supported by German Federal policy according to a report from 2011, concerned vertical site development/lifting systems, sanitary objects, housing entrance, and doors [8].

Thus, from an environmental gerontology perspective, providing HA/HM serves to support the desire of the vast majority of community-dwelling older adults to independently age in place as long as possible and thus to retain their living environments which have become meaningful over decades of housing [9,10,11]. Previous studies show strong links between living arrangements and later life disability among other factors, such as financial resources (e.g., [12]). In sum, there is considerable evidence for a positive relationship between accessible home environments and low rates of disability-related outcomes among community-dwelling elders, as well as on positive impacts of interventions through improvement of home environments to enhance outcomes such as performance of ADL, although there are mixed results with respect to fall-related outcomes [13]. Moreover, HA/HM may help to avoid or delay involuntary relocation to nursing homes or special care units [14]. Particularly the latter serves not only to fulfil personal needs and wishes in very old age but also to save money on a community or society level as well [15].

When HA/HM are recognized as public services, the state and the municipalities are responsible for funding these interventions through taxation. In other national contexts, citizens can choose to buy their HA/HM on the market, with tax deductions systems (when available) partially covering the expenses [16]. Sweden belongs to the former group of countries and here according to current legislation (SFS 1992:1574), HA grants are provided by the municipalities, covering the full costs of the interventions. The grant is provided irrespective of the applicant’s financial situation, and independently of whether the home is rented or owned. The annual expenditure for HA in Sweden is more than SEK 1 billion (around €115.5 million) and approximately 74,200 HAs are granted each year in Sweden [7].

In Germany, HA/HM is provided by the private sector and can be refunded up to a maximum amount per measure and year (currently up to €4000) from the German care insurance system [17]. Precise calculations on the numbers of needed dwellings, adapted or designed to be free from certain physical barriers, for the about 11 million older adults (65+) in Germany are hard to find. Given that only some of those older people who remain at home will need a completely barrier-free environment, the best estimations suggest that 2.5 million such dwellings will be needed by 2020. So far about 1.4 million mostly barrier-free dwellings are available in Germany, which leaves a supply gap of 1.1 million dwellings [18]. With respect to potential building/adaptation costs, this would mean that investments of about €39,000 million for adaptation or renovation are needed [19].

While basic preconditions for housing in later life with regard to cultural, financial, and social conditions differ substantially between—for example—Northern and Southern Europe and between Western and Eastern Europe, Germany and Sweden are relatively similar in this regard. A comparison between these countries with regard to housing policies therefore appear meaningful.

Data are generally difficult to find in countries where a public policy is lacking on HA/HM. On the contrary, in countries like Sweden data are available but the provision of HA as a legal right for all citizen makes it difficult to obtain estimates of the effectiveness of the intervention. Everyone in need in fact receives the grant and it is not feasible to identify a “control” group of older people not receiving the intervention [15]. Likewise, it is impossible to estimate the overall effect of HA policies on a societal level, using empirical data, even if such information could provide important insights to policy makers of countries where HA policies have not yet been set in place.

In order to address the issue of accessible housing for older people, this study will present mathematical simulations of policy changes with regard to housing accessibility that estimate the potential impact specifically on the instrumental activities of daily living (I-ADL), usage of home services, and related costs. The policy to be simulated is to provide funding to remove the five most severe environmental barriers in the homes of older people who are at risk of developing dependence in I-ADL. The models integrate empirical data from different settings with the aim of testing the hypothesis that the new policy removing potentially hindering housing features can maintain independence in I-ADL among older people, thus reducing the overall need for home services.

## 2. Materials and Methods

### 2.1. Theoretical Model

**Figure 1:**
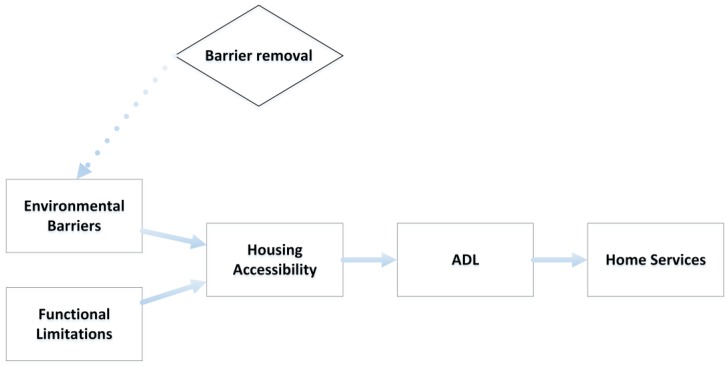
Theoretical model.

A key assumption of our model is that the degree of accessibility depends on the relation between barriers in the environment and the functional capacity of the individual (see Figure 1). Particular combinations of these two dimensions (barriers of the housing environment and functional capacity of the individual) may generate problems, such as the performance of ADL (both personal and instrumental), for those wishing to age in place [20]. When functional capacity decreases, there are specific demands on the design of the housing environment that need to be met, e.g., sufficient maneuvering space for individuals using different mobility devices, placement and design of controls and operable hardware that make them easy to reach and handle, etc. Theoretically, our model is underpinned by Lawton’s docility hypothesis, which states that those with lower functional capacity are more vulnerable to environmental demands, whereas those with higher functional capacity can withstand greater environmental demands [4].

In line with this theoretical model, we thus hypothesize that the degree of housing accessibility impacts on the ability to perform ADL activities independently, so that those with housing that is more inaccessible are at greater risk to become dependent in ADL, and that this in turn also affects the need of home services [21,22]. Dependence in any ADL is often the main criteria used to determine eligibility for paid home services, and Sandberg and colleagues [21] found that fewer than 3% of those receiving home services in Sweden were independent in all ADL activities. Therefore, we assumed that those simulated to become dependent in any ADL would then receive home services.

### 2.2. Data Sets and Variables

We used data from the German and Swedish samples of the EU-funded ENABLE-AGE survey study [9], which consisted of single living community dwelling people aged 80 to 89 years at baseline [2]. Participants were randomly drawn from official registers in their respective country, stratified by age (80–84, 85–89) and sex to guarantee sufficient sampling of 85+ (about 50%) and male (about 25%) persons. At baseline measurement (March 2002), there were 397 (41% of those invited) Swedish participants (75% women) and 450 (38% of those invited) German participants (78% women). At the 12-month follow-up in April 2003, the Swedish sample included 314 participants and the German sample included 322 participants. The main reasons for drop-out at follow-up were poor health (30%), lack of interest (25%), and death (20%). The data were collected by trained raters in the participants’ homes and included a combination of interviews and observations. The estimates for the simulations are based on those participants who completed both the baseline and 12-month follow-up. The policy to be simulated is focused on those who are still healthy (i.e., not dependent in ADL), and those who were lost to follow-up were less healthy. Therefore, we did not adjust the models for attrition.

Data on environmental barriers (that is, physical barriers in the home environment, such as high thresholds, narrow door openings, stairs without handrails, etc.), functional limitations (such as visual impairment, poor balance, reduced fine motor skills, etc.), and dependence on mobility devices was collected with the Housing Enabler instrument [23]. A case-specific measure of the magnitude of accessibility problems was obtained by combining data on the presence of environmental barriers (a checklist with 161 items) with an individual profile of functional limitations (a checklist with 14 items). Problematic combinations were assigned predefined scores on a scale from 1 to 4 (1 = potential problem; 2 = problem; 3 = severe problem; 4 = impossible problem) that were summed up for a total accessibility problem score. The total score predicts the magnitude of accessibility problems to arise in a specific case. For participants with no functional limitations or dependence on mobility devices, the housing accessibility problem score is always zero (i.e., no problems); higher scores indicate more accessibility problems. The theoretical maximum of the total score is 1844, while the contribution from each of the 161 environmental barriers theoretically ranges from 0 to 35.

ADL dependence was assessed using the ADL Staircase [24], which is comprised of five personal ADL (P-ADL) items (feeding, transferring, going to the toilet, dressing, and bathing) and four instrumental ADL (I-ADL) items (cooking, shopping, cleaning, and transportation). The ADL Staircase is administered using a combination of interview and observation. The sample used for this study was relatively healthy with all participants living independently in their own homes at the start of the study. Since very few participants were dependent on any of the P-ADL items at baseline (just 6% of the German sample and 7% of the Swedish sample), only the I-ADL data were used in this study. I-ADL was categorized using three levels of functioning for our models: dependent, independent, and independent with difficulty.

### 2.3. Simulation Methodology

To model the impact of a hypothetical policy for barrier removal in Sweden and Germany, we estimated the following parameters in a step-by-step procedure:

- the most prominent barriers in the two national housing stocks;
- the association of accessibility problem score and I-ADL dependence;
- the overall reduction of accessibility problems following the barrier’s removal;
- the cost of the barrier removal policy;
- the potential savings (in terms of home care provision cost) related to the policy impact.

#### 2.3.1. Step 1: Criteria to Prioritize Barriers for Removal

Assuming that it is not realistic to remove all possible barriers in the housing environment, we applied a procedure to select five prioritized barriers that would lead to the largest improvement in housing accessibility if removed for the entire at-risk population. Guiding criteria for this selection were:

1. the barriers should be located indoors or at entrances;
2. the barriers should have high impact on the range of potential accessibility problems generated, i.e., generating problems in relation to several functional capacities, such as vision, movement, handling and gripping etc., as shown by Slaug and colleagues [25];
3. the impact should also be potentially high in terms of severity, i.e., barriers more difficult to overcome, scoring at least 3 on the 1–4 scale;
4. the barriers should be prevalent in ordinary housing, i.e., occurring in at least 33% of the surveyed dwellings.

The barriers meeting all four criteria were ranked and the five that had the largest impact on the total accessibility problem score were selected.

#### 2.3.2. Step 2: Estimating the Association between Housing Accessibility Problems and I-ADL Dependence Using ENABLE-AGE Data

To estimate the impact of accessibility problems on I-ADL dependence, we utilized the baseline and one-year follow-up data from the ENABLE-AGE study. We first analyzed the independent effect of baseline accessibility problems (measured using the accessibility problem score) on the I-ADL at one-year follow-up by means of logistic regression models, adjusting for age, sex, and I-ADL dependence at baseline. That is, we used baseline housing accessibility problems to determine the increased risk of being dependent in each I-ADL after one year. The analysis was done for the German and Swedish samples separately. The level of significance was defined as *p* < 0.05. All analyses were performed using STATA (version 12.0, StataCorp, College Station, TX, USA) [26].

#### 2.3.3. Step 3: Simulating the Impact of Barrier Removal Policy Using ENABLE-AGE Data

To analyze the impact of barrier removal—and the reduced accessibility problem score thus achieved—on I-ADL dependence we compared the outcome in terms of number of persons being dependent in I-ADL for two different scenarios, the real data scenario and a simulated scenario based on the removal of the five prioritized barriers identified in Step 1. We used the odds ratios for the impact of the accessibility problem score on I-ADL dependence retrieved from the Step 2 logistic regression analyses (effect of a 1-point reduction in accessibility problem score) in order to calculate how many persons would have been dependent after one year in such a simulated scenario. We then calculated the “number needed to treat” [27] in order to have one more positive outcome (i.e., retained independence) than could be expected without any intervention.

#### 2.3.4. Step 4: Estimating Potential Costs of the New Barrier Removal Policy

We identified—as the ideal target population for our suggested barrier removal policy—older people living independently in the community with some difficulties, but not yet receiving any support service from the formal sector. In order to estimate the size of this population segment, we used the most recent figures available from official registers in Sweden [28] and Germany [29].

We first selected the same age ranges of population included in the ENABLE-AGE samples (80 to 90 years) and subtracted the estimated proportion of people living in assisted living. Thereafter, we estimated how many people were still independent but with some difficulty in I-ADL, as we assumed these to be those at highest risk of falling into dependence. These prevalences were estimated from ENABLE-AGE data. Finally, to define the target population size for our policy of barrier removal, we subtracted the proportion of those already receiving home services according to official statistics [30,31]. To estimate the cost for those at target for barrier removal, we multiplied the number of home adaptations implied with the average cost per home modification according to official statistics in Sweden [7]. For Germany, we did not have access to the actual costs of realized home modification/costs per home modification but instead used the maximum refund amount from the German care insurance system [19].

#### 2.3.5. Step 5: Estimating Potential Savings

To estimate the savings in terms of total costs for home services, we utilized data on the average number of hours per week that people receive home services in both countries. For Germany, we retrieved this data from the Medical Advisory Service of the German Associating of Statutory Health Insurance Funds [32], and from Sweden the data was from Official Statistics of Sweden, Social Welfare [30]. The average number of hours delivered during one year was then multiplied with the per-hour cost of home service, in order to get an annual cost. We calculated this cost for each of the four I-ADLs. As the same person may have been dependent in more than one I-ADL, we created a weight based on the number of estimated I-ADL dependencies per person to calculate total costs. Therefore, the total cost was calculated by summing individual I-ADL costs and multiplying the total by the weight.

### 2.4. Ethical Approval

All subjects gave their informed consent for inclusion before they participated in the ENABLE-AGE survey study. The study was conducted in accordance with the Declaration of Helsinki, and the protocol was approved by the Ethics Committee of Lund University (LU 324, 2002).

## 3. Results

### 3.1. Step 1: Barriers Prioritized for Removal

Twenty-six barriers fulfilled the four selection criteria in the Swedish sample, 21 in the German sample. From these barriers, we selected the five barriers in each national sample that would impact the housing accessibility (in terms of accessibility problem score reduction) most. The five barriers thus prioritized for removal are presented in Table 1. Three barriers were the same in each country. By systematically removing the top five barriers the average housing accessibility problem score in our study population changed from 148 to 134 in Germany and 148 to 132 in Sweden. On average, the accessibility problem scores for the German and Swedish samples were reduced by 14 and 16 points, respectively.

### 3.2. Step 2: Estimating the Relation between Accessibility Problem Score and I-ADL Dependence

The effect of a one-point change in housing accessibility on each of the I-ADL is shown in Table 2, for Germany and Sweden separately. All logistic regressions, except for “Cleaning” in Germany, showed that those with worse housing accessibility at baseline were more likely to become dependent in I-ADL one year later compared to those with better housing accessibility.

### 3.3. Step 3: Simulating the Impact of the Policy on I-ADL

Assuming a 14- and 16-point reduction in housing accessibility in the German and Swedish samples, respectively, and the odds ratios in Table 2, the simulated effect of the barrier removal policy is presented in Table 2 including the number needed to treat (i.e., number of barrier removal interventions needed to prevent one person from becoming dependent in I-ADL).

### 3.4. Step 4: Costs for Barrier Removal Policy

The size of the intervention target populations for the new barrier removal policy were 477,592 homes in Germany 51,536 homes in Sweden. For further details, see Table 3.

### 3.5. Step 5: Potential Savings

The potential reduction in costs for home services, when applying the results of the simulation analysis at the population level, is shown in Table 4. Note that the same person may be in more than one category of cases averted. In the German sample, people were on average dependent in 1.9 I-ADL, and in the Swedish sample the corresponding figure was 2.0 activities. These figures were used to calculate the weighted total cost reduction. In comparing the total costs for home modifications (Table 3) to the reduction in costs for home services (Table 4), we found that after 1.3 years in Sweden, and after 2.8 years in Germany, the initial cost for the new policy would have paid off.

## 4. Discussion

This study simulated the impact of policy changes in the area of housing accessibility, in terms of I-ADL dependency level, usage of home services and related costs. Our calculations integrated empirical data from the EU-funded ENABLE-AGE project in Sweden and Germany with publicly available statistics, testing the hypothesis that new policies to reduce potentially hindering housing features can help to maintain independence in I-ADL performance among older people who age in place. The main result of the simulation was that substantial numbers of cases of I-ADL dependence could be averted, thus reducing societal costs for home services.

However, an open question remains as to whether or not the reduction of costs can make up for the cost of barrier removals. Even if the costs for the new policy of barrier removals are large, it should be kept in mind that these are one-time costs, while costs for home services are likely to be repeated over time and potentially increase with deteriorating health. In our model, after a little more than one year in Sweden, and after two and half years in Germany, the initial cost for the new policy would have paid off, and after that date, an annual gain is likely to occur. Such gains can be higher the longer we enable persons to remain independent in I-ADL activities.

Our simulations show that improved accessibility of the ordinary housing stock has the potential to maintain or improve the health of our ageing population. Today, similar tools for preparing and making informed policy decisions are still largely lacking, whereas policy makers need such support to take decisions that are accurate and efficient. By developing simulation models based on the best knowledge available, this study provides policy makers with preliminary long-term predictions of the impact of housing adaptation policies.

There are, however, significant study limitations that have to be considered. The effect of barrier removal on accessibility problems is based on ENABLE-AGE data, but also on theoretical assumptions with respect to this relationship. While the ENABLE-AGE samples were randomly selected they were only recruited regionally around the study sites in each country. We therefore, cannot be certain that they are truly representative of the national population of single living people aged 80–90 years of either country. While it was not possible to weight the ENABLE-AGE data to simulate a national sample due to the sampling frame, we did use national statistics to estimate the proportions of people living independently who would be recipients of the policy intervention. The one-year follow-up period causes additional limitations. The association between housing accessibility and I-ADL may be biased toward a healthier sample as those who were sicker were less likely to complete the follow-up. However, we expect that if the sicker participants had been able to complete the follow-up, it would have actually led to a larger effect, and therefore, we believe our estimates are likely more conservative. Moreover, keeping in mind that we only have a one year follow-up, we do not know for how long the positive effects will be stable beyond this time frame. That is, for how long can I-ADL dependence be delayed?

The ageing population in combination with the deficiencies in the ordinary housing stock thus calls for large-scale measures [35]. However, for such measures to be adequate, accurate, and cost-effective, they have to be based on the best possible knowledge about the ordinary housing stock, the functional status of the population and the costs and feasibility of different housing adaptations [20]. A housing stock that can accommodate a population aging in place requires long-term planning and plausible projections of development 20–30 years into the future [36]. Hence, future studies need to examine the dynamics of aging in place and I-ADL independence over longer periods to further substantiate the evidence presented in this study for the benefit of new housing accessibility policies.

An ordinary housing stock that can accommodate an ageing population is of utmost importance from a public health perspective. The present simulations did not take into account the potential reductions in costs related to maintenance of health or informal care. A housing environment that is not accessible also increases the risk for a lower degree of social participation, which can turn into poorer self-management, isolation, and ultimately higher health care needs [37]. Furthermore, this study only evaluated the policy impact on paid home services. As many services are provided by informal carers (usually a family member) [38], future simulations should also evaluate the impact of housing accessibility policy related to the burden of informal care, such as lost wages, physical and mental health, etc.

## 5. Conclusions

Keeping the study limitations in mind, our findings suggest that a policy that provides funding to remove the most severe environmental barriers in the homes of older people who are at risk of developing dependence in I-ADL, can contribute to positive effects in terms of maintaining I-ADL independence among community-dwelling older people. Moreover, considering a time perspective, our findings suggest such a policy change can achieve a reduction of societal burden in terms of provision of home services. Such models can provide policy makers with the tools needed to make informed decisions when weighing future policy options to enhance quality of life while minimizing costs.

## Figures and Tables

**Table 1:** Step 1: barriers prioritized based on their relevance for housing accessibility.

Germany	Sweden
**1. Wall-mounted cupboards and shelves placed extremely high in the kitchen**	**1. Wall-mounted cupboards and shelves placed extremely high in the kitchen**
**2. Doors at entrance that do not stay in open position/close quickly**	2. No grab bars at shower/bath and/or toilet
**3. Insufficient maneuvering areas in the kitchen**	**3. Doors at entrance that do not stay in open position/close quickly**
4. Heavy doors without automatic opening at entrance	**4. Insufficient maneuvering areas in the kitchen**
5. Very high, very low, and/or irregular heights of risers at entrance stair	5. High thresholds and/or steps at the entrance

Note: Barriers that are common for both countries are marked with bold letters.

**Table 2:** Steps 2 and 3: simulated scenarios of absolute risk of becoming dependent in I-ADL, averted cases and number needed to treat.

I-ADL Type by Country	a1. Cases of I-ADL Dependence Baseline *n* (%)	a2. Cases of I-ADL Dependence: Follow Up *n* (%)	* b. Risk of Dependence in I-ADL or (95% CI)	c. Cases of I-ADL Dependence—Simulated Intervention Effect	d. Absolute Risk Reduction (c–a2, %)	f. Number Needed to ”Treat”
**Germany (*n* = 322)**						
Cooking	56 (17.5%)	64 (19.9%)	1.004 (1.000 to 1.008)	60 (18.7%)	1.2%	86
Shopping	118 (36.8%)	150 (46.6%)	1.003 (1.000 to 1.007)	143 (44.3%)	2.2%	45
Cleaning	141 (44.1%)	143 (44.4%)	1.001 (0.998 to 1.005)	141 (43.6%)	0.8%	130
Transportation	44 (14.7%)	80 (25.7%)	1.005 (1.001 to 1.008)	75 (24.0%)	1.7%	58
**Sweden (*n* = 314)**						
Cooking	54 (17.2%)	65 (20.7%)	1.005 (1.002 to 1.009)	59 (18.9%)	1.8%	56
Shopping	64 (20.4%)	88 (28.0%)	1.004 (1.001 to 1.008)	82 (26.0%)	2.0%	50
Cleaning	95 (30.4%)	122 (38.9%)	1.011 (1.007 to 1.015)	100 (32.0%)	6.8%	15
Transportation	127 (40.4%)	125 (39.9%)	1.004 (1.001 to 1.007)	117 (37.4%)	2.5%	39

* Risk of becoming dependent in I-ADL after one year is significantly associated with baseline housing accessibility except for “Cleaning” in Germany. Logistic regressions were adjusted for sex, age, and baseline I-ADL score. Note: The number of valid cases in the simulated scenario may differ slightly from the baseline scenario, as it is dependent on valid data for all variables used to calculate the odds ratios.

**Table 3:** Step 4: estimating the population to target for intervention and the costs to implement the barrier removal policy.

Sub-Steps to Calculate Policy Cost	Germany	Sweden
**Target Population**		
Total population age 80–90 *	3,740,395 ^a^	427,940 ^b^
Estimated proportion living in assisted living	9% ^a^	14% ^c^
Number of people living in private homes	3,399,072	368,028
Number of people independent in I-ADL but with difficulty (estimation from ENABLE-AGE samples)	1,142,088 (34%)	134,330 (37%)
Number currently receiving home services	664,496 ^a^	82,774 ^c^
Number to target for barrier removal policy	477,592	51,556
**Home modifications**		
Number of home modifications	477,592	50,913
Cost per home modification (Euro)	2557 ^d^	1570 ^e^
Total Cost (Euro)	1,221,203,235	79,933,207

* Age 80–89 for Germany; ^a^ From Pflegestatistik 2013, Statistisches Bundesamt [31]; ^b^ From Statistics Sweden, as of 31 December 2014 [28]; ^c^ From Statistics—Social Welfare [30]; ^d^ Maximum refund amount from German care insurance system [19]; ^e^ Average cost of government funded home modification in Sweden [7].

**Table 4:** Step 5: estimated savings from the reduction of costs for home services.

I-ADL Dependence	Cases Averted (95% CI)	Reduction of Hours/Week ^a^ (95% CI)	Reduction of Cost/Year (Euro) ^b^ (95% CI)
**Germany**			
Shopping	10,684 (701 to 20,694)	138,886 (9111 to 269,026)	312,065,586 (20,470,775 to 604,479,229)
Transportation	8275 (2011 to 14,563)	107,571 (26,138 to 189,316)	241,702,602 (58,730,065 to 425,378,494)
Cooking	5555 (367 to 10,765)	72,215 (4768 to 139,939)	162,261,974 (10,713,949 to 314,431,098)
Cleaning	3673 (−6319 to 13,695)	47,751 (−82,145 to 178,032)	107,291,727 (−184,572,996 to 400,023,804)
Weighted total			434,362,835 (−49,939,164 to 920,253,167)
**Sweden**			
Shopping	1035 (314 to 1757)	8083 (2456 to 13,725)	19,241,787 (5,847,783 to 32,674,489)
Transportation	1313 (199 to 2432)	10,262 (1552 to 19,000)	24,429,313 (3,694,727 to 45,231,298)
Cooking	914 (269 to 1562)	7143 (2104 to 12,202)	17,005,439 (5,008,795 to 29,045,992)
Cleaning	3531 (2282 to 4784)	27,585 (17,831 to 37,373)	65,667,948 (42,449,297 to 88,970,055)
Weighted total			62,662,789 (28,270,459 to 97,170,909)

^a^ Germany: Average of 13 h per week [32]; Sweden: Average of 8 h per week [30]; ^b^ Germany: 43 Euro per hour cost of home services [33]; Sweden: 47 Euro per hour cost of home services [34]; adjusted for inflation 2012.
